# Immediate effects of first-line thrombectomy devices for intracranial atherosclerosis-related occlusion: stent retriever versus contact aspiration

**DOI:** 10.1186/s12883-020-01862-6

**Published:** 2020-07-18

**Authors:** Joonsang Yoo, Seong-Joon Lee, Jeong-Ho Hong, Yong-Won Kim, Ji Man Hong, Chang-Hyun Kim, Dong-Hun Kang, Jin Wook Choi, Yong-Sun Kim, Sung-Il Sohn, Yang-Ha Hwang, Jin Soo Lee

**Affiliations:** 1 Department of Neurology, Keimyung University School of Medicine, Daegu, South Korea; 2 Department of Neurology, National Health Insurance Service Ilsan Hospital, Goyang, South Korea; 3 Department of Neurology, Ajou University Medical Center, Ajou University School of Medicine, 164 World cup-ro, Yeongtong-gu, Suwon, 16499 South Korea; 4 Department of Neurology, Kyungpook National University Hospital, School of Medicine, Kyungpook National University, 130 Dongdeok-ro, Jung-gu, Daegu, 41944 South Korea; 5 Department of Neurosurgery, Keimyung University School of Medicine, Daegu, South Korea; 6 Department of Neurosurgery, Kyungpook National University School of Medicine, Daegu, South Korea; 7 Department of Radiology, Ajou University School of Medicine, Suwon, South Korea; 8 Department of Radiology, Kyungpook National University School of Medicine, Daegu, South Korea

**Keywords:** Ischemic stroke, Reperfusion therapy, Stent retriever, Contact aspiration, Intracranial atherosclerosis, Endovascular treatment

## Abstract

**Background:**

Although stent retriever (SR) is recommended as a frontline device of endovascular treatment (EVT) for embolic large artery occlusion causing acute ischemic stroke, contact aspiration (CA) device showed similar efficacy in the recent trials. However, the efficacy of the both devices as first-line therapy for intracranial atherosclerotic stenosis (ICAS)-related large vessel occlusion has not yet been established. Therefore, we compared the immediate effects and final outcomes of SR and CA as first-line devices for treating ICAS-related occlusions.

**Methods:**

We retrospectively analyzed the data of patients who underwent EVT for acute ischemic stroke from the registry of three Korean hospitals. Patients with ICAS-related occlusion who were treated within 24 h of onset of the symptoms were included. We investigated immediate reperfusion performance, immediate safety outcomes, and 3-month clinical outcomes for the two first-line devices.

**Results:**

Of the 720 registered patients, 111 were eligible for this study. Forty-nine patients (44.1%) used SR and 62 (55.9%) used CA as the first-line device. Achieving successful reperfusion immediately after first-line thrombectomy was more frequent in the SR group than that in the CA group (77.6% vs. 43.5%, *p* = 0.001), with fewer additional rescue treatments (12.2% vs. 59.7%, *p* < 0.001). The incidence of iatrogenic dissection or rupture was lower in the SR group than that in the CA group (8.2% vs. 29.0%, *p* = 0.012). After additional rescue treatments, however, the final successful reperfusion rate did not differ between the two groups (SR 87.8% vs. CA 77.4%, *p* = 0.247), and there was no significant difference in the 3-month good outcomes (modified Rankin Scale, *p* = 0.524).

**Conclusions:**

First-line SR thrombectomy showed higher immediate reperfusion and less vessel injury for ICAS-related occlusions than CA. However, there was no significant difference in the final reperfusion status or 3-month outcomes from additional rescue treatments.

## Background

Endovascular treatment (EVT) has shown favorable results in major trials mainly using stent retriever (SR) [1]. Therefore, SR is recommended as a first-line device for treatment of large vessel occlusion in the AHA/ASA guidelines [2]. However, contact aspiration (CA) showed outcomes comparable to those with SR in recent studies [3, 4].

Intracranial atherosclerotic stenosis (ICAS)-related large vessel occlusion (ICAS-LVO) is a common cause of stroke, especially among the Asian population [5–9]. In ICAS-LVO, immediate reocclusion occurs frequently, requiring further rescue treatment, and the final reperfusion rate tends to be lower than that of embolic occlusions [10, 11]. Because of these features, a different approach may be needed for ICAS-LVO than that for general embolic occlusions [12].

However, which device is suitable for first-line therapy in ICAS-LVO is unclear, because most of the previous randomized trials were based on Western populations. Knowledge about which device is more effective in ICAS-LVO would be useful in selecting a more suitable device for patients with ICAS-LVO before EVT [13–15]. Recently, a study comparing outcome of SR and CA in ICAS-LVO patients at two hospitals was published [16]. However, studies on immediate performances and side effects are still insufficient. Therefore, we compared the immediate effects and final outcomes of using SR or CA as first-line devices in the treatment of ICAS-LVO.

## Methods

### Study population and inclusion criteria

All clinical and image data were de-identified and allocated study identification numbers. The protocol for data collection was approved by the Institutional Review Board of each hospital. Our study was implemented in accordance with the ethical standards of the 1964 Declaration of Helsinki and its later amendments. The need for written informed consent was waived because of the retrospective nature of this study. The data of this study are available from the corresponding author upon reasonable request.

This was a retrospective analysis of the ASIAN KR (Acute Stroke due to Intracranial Atherosclerotic occlusion and Neurointervention Korean Retrospective) registry. The details of the registry were previously published [11]. In brief, the registry consists of data on consecutive patients who underwent emergency EVT for cervicocerebral artery occlusions causing acute ischemic stroke at three stroke centers in Korea. The patients were enrolled between January 2011 and February 2016. For the current study, the inclusion criteria were: 1) patients with intracranial large artery occlusions; 2) underlying etiology classified as ICAS; and 3) time from symptom onset to EVT start ≤24 h.

### Classifying ICAS-LVO

Underlying ICAS, which should be differentiated from embolism or other etiologies, was defined based on the remaining fixed focal stenosis during EVT. The step-by-step evaluations for differentiation were described previously [11]. Briefly, after confirmation of arterial occlusion, patients with uncommon stroke etiologies, such as dissection, Moyamoya disease, and vasculitis were excluded. Embolic occlusion was classified based on complete vessel recanalization after thrombectomy, and ICAS-related occlusion was classified when a remnant stenosis of > 70%, or a lesser degree of stenosis with a tendency toward reocclusion and/or flow impairment after thrombectomy was observed [11, 17]. This classification was further confirmed by repeat angiography during admission.

### EVT procedure

Devices were selected at the discretion of neuro-interventionists based on the consensus within each stroke team. In the current study, we divided patients into two groups based on whether SR or CA was used as the first-line device. Solitaire AB/FR (Medtronic, Irvine, CA, USA) or Trevo (Stryker, Kalamazoo, MI, USA) belonged to the SR group, and 1st generation or 2nd generation Penumbra MAX systems (Penumbra Inc., Alameda, CA, USA) belonged to the CA group. Balloon guide catheters, adjuvant local lytic infusion, intracranial angioplasty and/or stenting were implemented as needed.

### Image and clinical assessment

Premorbid functional status, conventional vascular risk factors, and laboratory findings assessed during admission were collected. Stroke severity was assessed using the initial National Institutes of Health Stroke Scale (NIHSS) score. Clinical outcomes were measured using the modified Rankin Scale (mRS) score at 3-months. mRS scores 0 to 2 or no change between premorbid and 3-month mRS were considered as good outcomes.

The location of initial occlusion site was determined using baseline computed tomography angiography or magnetic resonance angiography. Reperfusion performance was evaluated using modified treatment in cerebral ischemia (mTICI) grade [18]. Successful reperfusion was defined as mTICI grade 2b or higher. When the first-line device did not achieve the desired reperfusion status, rescue treatments were allowed, including other thrombectomy devices, fibrinolytics, angioplasty, and intracranial stenting. The number of EVT methods used until achievement of final reperfusion was also determined. Intracerebral hemorrhages were classified in accordance with the European Cooperative Acute Stroke Study criteria [19]. Subarachnoid hemorrhage (SAH) was classified using the modified Fisher scale [20].

### Primary outcomes

Immediate reperfusion performance as a primary efficacy outcome was assessed immediately after the first attempt and full use of the first-line thrombectomy device, and final reperfusion status was assessed at the last angiography. We also assessed the immediate side effects, such as 1) vasospasm, 2) iatrogenic dissection or rupture by thrombectomy, and 3) new embolism in other vessels as primary safety outcomes. Angiographic lesions with surface irregularity or intimal flap were considered as iatrogenic vessel injuries.

### Statistical analysis

Variables are expressed as mean ± standard deviations, medians (interquartile ranges), or numbers (percentages), as statistically appropriate. We compared baseline characteristics, as well as clinical and imaging results between the SR and CA groups using chi-squared tests and independent Student’s *t*-tests or Wilcoxon rank-sum tests, respectively. To determine the factors associated with good clinical outcome and iatrogenic dissection, we performed multivariate analyses after adjusting for age, sex, and variables with *p* < 0.1 in the univariate analysis. *p* values were two-tailed, and variables were considered significant at *p* < 0.05. All statistical analyses were performed with R version 3.5.1 (http://www.R-project.org).

## Results

### Baseline characteristics

**Fig. 1:**
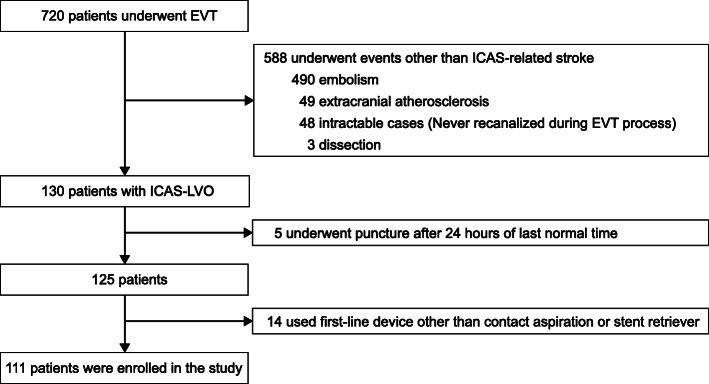
Flowchart of the current study. EVT: Endovascular treatment; ICAS: Intracranial atherosclerotic stenosis; ICAS-LVO: ICAS-related large vessel occlusion

During the study period, 111 of the 720 registered patients were included in the current study (Fig. 1). Mean age of the included patients was 65.2 ± 13.3 years, and 70 patients (63.1%) were men. Among them, 49 patients (44.1%) belonged to the SR group, and 62 (55.9%) to the CA group. Demographics and baseline characteristics of the patients between two groups did not differ (Table 1).

**Table 1:** Baseline characteristics of patients with intracranial atherosclerotic stenosis (ICAS)-related occlusions and endovascular treatments

	Contact aspiration (*n* = 62)	Stent retriever (*n* = 49)	*P*
Demographics			
Age, years	66.3 ± 11.7	63.9 ± 15.2	0.348
Sex, men	39 (62.9)	31 (63.3)	> 0.999
Risk factors			
Hypertension	38 (61.3)	33 (67.3)	0.645
Diabetes mellitus	20 (32.3)	14 (28.6)	0.833
Dyslipidemia	23 (37.1)	12 (24.5)	0.225
Atrial fibrillation	14 (22.6)	10 (20.4)	0.965
Smoker	26 (41.9)	13 (25.5)	0.103
Medications prior admission			
Antiplatelets	11 (17.7)	7 (14.3)	0.817
Anticoagulants	2 (3.2)	4 (8.2)	0.403
Initial occlusion site			0.078
Internal carotid artery	12 (19.4)	2 (4.1)	
Middle cerebral artery, M1	36 (58.1)	36 (73.5)	
Middle cerebral artery, M2	2 (3.2)	1 (2.0)	
Vertebral artery	2 (3.2)	4 (8.2)	
Basilar artery	10 (16.1)	6 (12.2)	
Initial NIHSS score	16.5 (11–22)	15 (12–19)	0.340
ASPECTS score^a^	7.5 (5–9)	7 (6–8)	0.625
Laboratory findings			
Hemoglobin, g/dL	14.0 ± 1.8	13.9 ± 2.0	0.764
White blood cells, ×10^9^/L	9.6 ± 3.6	11.5 ± 5.6	0.047
Platelets, ×10^9^/L	240 ± 77	239 ± 59	0.928
Glucose, mmol/L	8.2 ± 3.3	8.4 ± 3.3	0.813
Intravenous tPA	27 (43.5)	23 (46.9)	0.869
Use of balloon guide catheter	36 (58.1)	26 (53.1)	0.738
Onset to door time, min	208 (111–387)	172 (105–560)	0.801
Door to puncture time, min	97.5 (77–127)	111 (92–138)	0.106
First-line thrombectomy device			
Solitaire		41 (83.7)	
Trevo		8 (16.3)	
Penumbra, 1st generation	45 (72.6)		
Penumbra, 2nd generation	17 (27.4)		

*ICAS* Intracranial atherosclerotic stenosis; *NIHSS* National Institutes of Health Stroke Scale; *ASPECTS* Alberta Stroke Program Early CT Score; *tPA* tissue plasminogen activator

^a^Baseline ASPECTS on quality imaging was evaluated in 81 patients (91.0% patients with anterior circulation infarction)

### Immediate effects following first-line thrombectomy

**Fig. 2:**
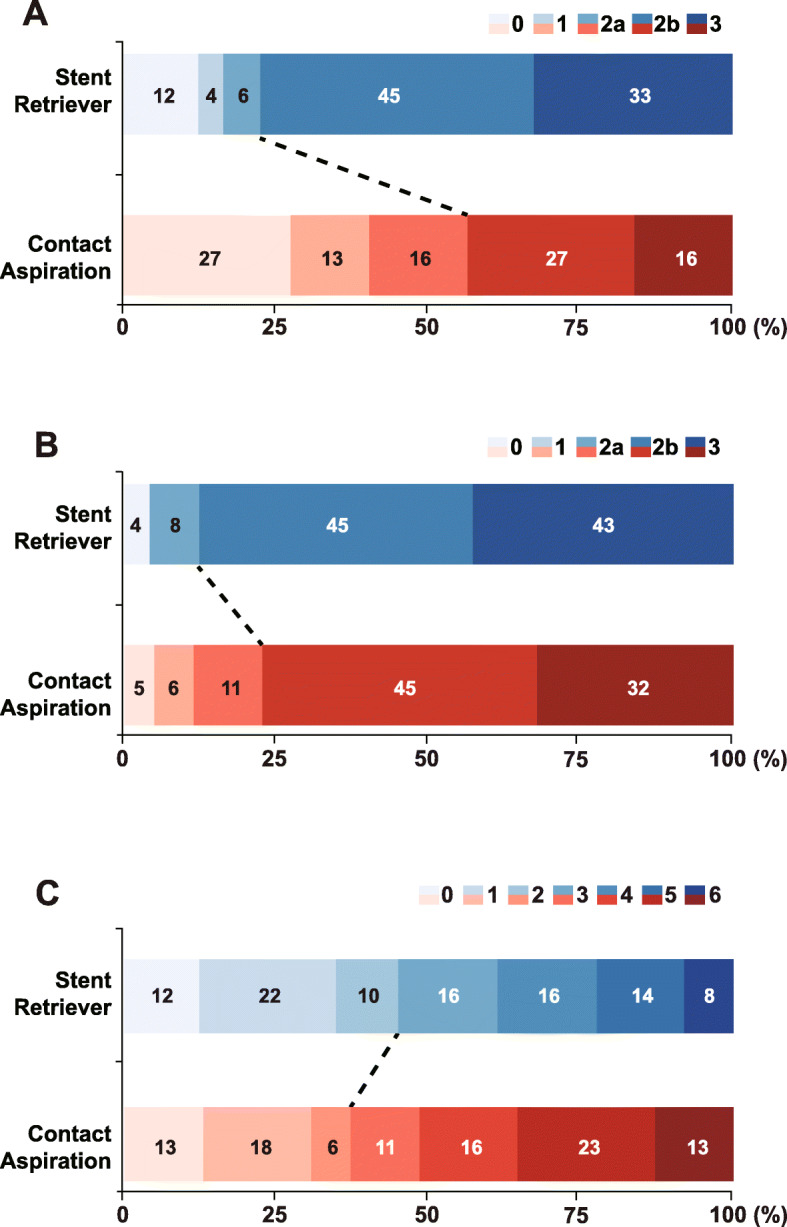
Treatment outcomes. mTICI grade **a** immediately after first-line thrombectomy and **b** of final thrombectomy. **c** Modified Rankin Scale at 3 months. mTICI: modified thrombolysis in cerebral infarction

Table 2 summarizes the comparative results regarding treatment. Successful reperfusion after first attempt of thrombectomy was not significantly different between the two groups (SR 28.6% vs. CA 17.7%, *p* = 0.260). Immediate successful reperfusion after first-line thrombectomy was achieved more frequently in the SR group (SR 77.6% vs. CA 43.5%, *p* = 0.001) (Fig. 2a). Transient vasospasm occurred in four patients in the SR group (SR 8.2% vs. CA 0%, *p* = 0.035). In contrast, iatrogenic dissection or rupture occurred more frequently in the CA group (SR 8.2% vs. CA 29.0%, *p* = 0.012). Odds ratio of iatrogenic dissection or rupture in the CA group was 4.488 in a logistic regression analysis after adjusting age, sex, presence of atrial fibrillation, initial NIHSS score, procedural time, and total number of EVT methods (95% confidence interval [CI] 1.394–17.676, *p* = 0.018) (Supplementary Table 1). This rate of iatrogenic dissection or rupture was not different between the first-generation Penumbra and Penumbra MAX or between the Solitaire and Trevo stents; however, these results must be interpreted with caution owing to the small sample size. The frequency of new embolisms at other vessels was relatively rare and did not differ between the groups (SR 6.1% vs. CA 3.2%, *p* = 0.653).

**Table 2:** Radiologic and clinical outcomes after endovascular treatment

	Contact aspiration (n = 62)	Stent retriever (n = 49)	*P*
***Immediate effects following first-line thrombectomy***			
Successful reperfusion after first attempt of thrombectomy	11 (17.7)	14 (28.6)	0.260
Successful reperfusion after first-line thrombectomy	27 (43.5)	38 (77.6)	0.001
Immediate side effect by first-line thrombectomy			
Vasospasm	0	4 (8.2)	0.035
Iatrogenic dissection or rupture	18 (29.0)	4 (8.2)	0.012
New embolism in other vessels	2 (3.2)	3 (6.1)	0.653
***Rescue treatments after first-line thrombectomy***			
Switching to the other device	37 (59.7)	6 (12.2)	< 0.001
Tirofiban infusion	29 (46.8)	23 (46.9)	> 0.999
Balloon angioplasty	7 (11.3)	5 (10.2)	> 0.999
Permanent intracranial stenting	6 (9.7)	6 (12.2)	0.901
***Final endovascular treatment results***			
Final successful reperfusion	48 (77.4)	43 (87.8)	0.247
Total number of endovascular techniques	2 (2–3)	2 (1–2)	0.003
Puncture to final angiography time, min	76 (48–116)	63 (45–92)	0.151
***Imaging and clinical outcomes after endovascular treatment***			
Hemorrhagic complication			
Any intracerebral hemorrhagic transformation	16 (25.8)	10 (20.4)	0.659
Parenchymal hemorrhage, type 2	2 (3.2)	4 (8.2)	0.403
SAH, grade 3 or 4	0	2 (4.1)	0.193
Good outcome at 3 months	23 (37.1)	22 (44.9)	0.524

*SAH* Subarachnoid hemorrhage

### Rescue treatments after first-line thrombectomy

The rate of switching to the other thrombectomy device (from SR to CA / from CA to SR) for rescue treatment was significantly lower in the SR group (SR 12.2% vs. CA 59.7%, *p* < 0.001). As other rescue treatments, tirofiban infusion (SR 46.9% vs. CA 46.8%, *p* > 0.999), balloon angioplasty (SR 10.2% vs. CA 11.3%, *p* > 0.999), and permanent intracranial stenting (SR 12.2% vs. CA 9.7%, *p* = 0.901) were performed in both groups with no significant differences in their rates.

### Final endovascular treatment results

Final successful reperfusion rate was similar between the two groups (SR 87.8% vs. CA 77.4%, *p* = 0.247) (Fig. 2b). However, the endovascular techniques were fewer in the SR group (SR 2 [1, 2] vs. CA 2 [2, 3], *p* = 0.003). Puncture to final angiography time was not significantly different (SR 76 [48–116] min vs. CA 63 [45–92] min, *p* = 0.151).

### Imaging and clinical outcomes after endovascular treatment

Intracerebral hemorrhagic transformation of any type (*p* = 0.659) and parenchymal hematoma type 2 (*p* = 0.403) occurred at similar rates in the two groups. Thick subarachnoid hemorrhages occurred in two patients in the only SR group. There was no significant difference in good clinical outcome at 3 months (SR 44.9% vs. CA 37.1%, *p* = 0.524) (Fig. 2c). In multivariate analysis, 3-month clinical outcome was not associated with the first-line thrombectomy device (Table 3). Instead, age, initial stroke severity, initial occlusion site and final successful reperfusion were independently associated with good clinical outcome at 3-months.

**Table 3:** Factors associated with good clinical outcome in patients with intracranial atherosclerotic stenosis (ICAS)-related occlusion

	Univariate analysis			Multivariate analysis	
	Poor outcome (*N* = 66)	Good outcome (*N* = 45)	*P*	Odds ratio (95% CI)	*P*
Demographics					
Age, years	68.7 ± 11.9	60.2 ± 14.2	0.001	0.956 (0.915–0.995)	0.035
Sex, men	39 (59.1)	31 (68.9)	0.395	0.863 (0.287–2.542)	0.789
Risk factors					
Hypertension	46 (69.7)	25 (55.6)	0.186		
Diabetes mellitus	20 (30.3)	14 (31.1)	> 0.999		
Dyslipidemia	22 (33.3)	13 (28.9)	0.774		
Atrial fibrillation	12 (18.2)	12 (26.7)	0.406		
Smoker	20 (30.3)	19 (42.2)	0.276		
Initial occlusion site			0.001		
Internal carotid artery	13 (19.7)	1 (2.2)		Ref	
Middle cerebral artery	36 (54.5)	39 (86.7)		12.544 (1.777–259.986)	0.030
Vertebral/Basilar artery	17 (25.8)	5 (11.1)		5.021 (0.520–117.656)	0.206
Initial NIHSS score	19 (14–23)	12 (9–16)	< 0.001	0.830 (0.742–0.913)	< 0.001
ASPECTS score^a^	7 (4–8)	8 (6–10)	0.005		
Onset to door time, min	196 (120–421)	204 (82–457)	0.833		
Door to puncture time, min	106 (81–130)	107 (89–123)	0.881		
IV tPA	30 (45.5)	20 (44.4)	> 0.999		
Stent retriever as first-line device	27 (40.9)	22 (48.9)	0.524	0.987 (0.346–2.770)	0.980
Primary successful reperfusion	35 (53.0)	30 (66.7)	0.217		
Final successful reperfusion	51 (77.3)	40 (88.9)	0.190	5.479 (1.441–24.841)	0.018
Number of techniques	2 (2–3)	2 (1–3)	0.457	1.208 (0.643–2.293)	0.555
Laboratory findings					
Hemoglobin, g/dL	14.0 ± 1.9	14.0 ± 2.0	0.917		
White blood cells, ×10^9^/L	11.2 ± 5.0	9.4 ± 3.8	0.036	0.910 (0.784–1.039)	0.186
Platelets, ×10^9^/L	242 ± 72	236 ± 65	0.616		
Glucose, mmol/L	8.2 ± 3.1	8.4 ± 3.5	0.709		

^a^Baseline ASPECTS on quality imaging was evaluated in 81 patients (91.0% patients with anterior circulation infarction)

*NIHSS* National Institutes of Health Stroke Scale; *ASPECTS* Alberta Stroke Program Early CT Score; *IV tPA* Intravenous tissue plasminogen activator

## Discussion

In the current study, we investigated if there were differences primarily in immediate reperfusion performance and side effects, and secondarily in post-procedural hemorrhagic complications and clinical outcomes, depending on the choice of the first-line devices between SR and CA in patients with ICAS-LVO. Based on the results of our retrospective analysis of a multicenter registry, we could cautiously mention that the use of SR may be safer and more effective for ICAS-LVO than CA as a first-line thrombectomy method in terms of immediate reperfusion success and immediate occurrence of iatrogenic dissection or rupture.

Recently, studies have reported comparisons of reperfusion performance or side effects of CA and SR when performing EVT in overall LVO patients. In the ASTER trial, the reperfusion success did not differ between CA and SR (mTICI score of 2b or 3, SR 67.7% vs. CA 63.0%) [3, 21]. More recently, the COMPASS trial showed that first-line CA treatment was not inferior to first-line SR treatment with respect to good outcomes (mRS 0–2, SR 50% vs. CA 52%;non-inferiority margin absolute difference 15%; *p*_non-inferiority_ = 0.0014) [4]. The secondary angiographic outcomes did not significantly differ between groups but tended to be better in CA rather than in SR in terms of the median time to successful reperfusion (SR 33 min vs. CA 22 min, *p* = 0.019). These trials provide convincing evidence that CA may be used as an alternative to SR thrombectomy as first-line therapy in anterior circulation LVO-related acute ischemic stroke within 6 h of symptoms onset. However, these trials were performed in the Western countries (France, USA, and Canada), where the most predominant cause of emergency LVOs was known to be embolic occlusion. Large artery atherosclerosis as the suspected cause of stroke accounted for around 7.9% in the ASTER trial [3], and 5.5% was reported in a retrospective study from France [22]. In contrast, ICAS as an underlying etiology of LVO is reported to be more frequent among the Asian populations (17.6–19.0% based on most relevant methodology) [9, 11]. Mechanical thrombectomy in a severe atherosclerotic arterial bed may have different performance and side effects because stenotic lesions can cause additional friction with devices [23]. It has been suggested that ICAS-LVO may require a different approach than embolic LVO [8, 9]. Therefore, we evaluated if there were differences between SR and CA in terms of performance and side effects after first-line thrombectomy.

In the current study, immediate reperfusion performance was better when SR was used as a first-line device in ICAS-LVO. Therefore, rescue treatment involving the use of other devices was more common in the CA group, and the overall number of techniques was also higher with the use of CA. These results are consistent with those of the ASTER trial, in which rescue treatment after fist-line strategy tended to be more frequent in the CA group (32.8%) than in SR group (23.8%) with marginal statistical significance (3), and in a previously reported observational comparative study (CA 45.2% vs. SR 13.5%) that showed statistical significance [24]. In the current study, as rescue techniques, the rates of tirofiban infusion, angioplasty, or stent insertion were similar between the two groups, whereas the switching rate from CA to SR was significantly higher in the CA group than that in the SR group. Although the successful reperfusion rate was higher in the SR group, there were no significant differences in rescue techniques such as tirofiban infusion, suggesting that ICAS-LVO tends to reocclusion and still requires additional treatment in many patients.

Among the immediate side effects, the frequency of iatrogenic dissection or rupture appeared to be prominent. Mechanical thrombectomy may cause vessel damage [25–28]. Several animal studies have shown occurrence of intima and medial damage after thrombectomy using SR or CA devices. Some of the vessel damage could be transient, but some could leave long-term damage [26]. Despite these natural characteristics of mechanical thrombectomy, the occurrence rate of iatrogenic dissection or rupture in both groups of the current study was higher than that that of recent trials reported in Western countries. In the ASTER trial, the frequency of arterial dissection was only 1.1% (SR group) to 2.6% (CA group) (3). More frequent vessel injury in our study might be caused by vessel stenosis. When outcomes were compared between ICAS- and embolic LVOs with Solitaire stent thrombectomy, the frequency of vessel injury using the same definition as the current study accounted for 13.5% vs. 3.7%, respectively [29]. In addition, immediate vessel injury was more frequent in the CA group than in SR group in the current study. In general, SR is expected to have more vessel injury than CA. However, ICAS can make the tip of CA catheter more difficult to face thrombus due to hurdle-like anatomy of stenotic lumens. A recent review article attributes this phenomenon to the possibility that the tip of aspiration catheter may not properly contact the in situ thrombi but may face the surface of ICAS [30].

Even though rescue treatments were more frequently used and procedural time was longer in the CA group than in SR group, final reperfusion success and 3-month good outcome did not differ. Taken together, SR can be considered more advantageous as a first-line device than CA for treatment of ICAS-LVO. However, despite this immediate advantage, there was no significant difference in the outcome following aggressive rescue treatment and device switching [31, 32].

Despite of the differences of immediate reperfusion performance and side effect, there was no difference in the final outcome. The difference in the outcome of the first-line treatment may be complemented by the rescue treatment, but the effect of post-procedure management cannot be denied. Because the purpose of this study is not related to this, we could not present relevant data, but the followings could be considered. While lowering blood pressure when reperfusion after embolic occlusion is recommended to prevent reperfusion injury, maintaining blood pressure slightly higher for remnant stenosis would be helpful to maintain cerebral perfusion pressure in patients with ICAS. In addition, as shown in SAMMPRIS trial, we should consider maintaining sufficient antithrombotic activity with dual antiplatelets at least the first 3 months and use of intensive statin in anticipation of regression of stenosis [33, 34]. Further studies are needed on the post-procedure management with ICAS patients.

This study has several limitations. First, this was a retrospective study; therefore, it is not free from selection bias. Second, patients who were enrolled early in the study period in the registry underwent treatment with outdated devices, such as the first-generation Penumbra that are not currently in use. Moreover, new CA devices have been commercialized recently, and further studies on their effects and safety are needed. To this point, we also compared the first-generation and subsequent Penumbra systems but there was no difference in terms of outcomes (data not shown). In addition, future research may be required as new techniques such as Solumbra or stent retriever assisted vacuum-locked extraction have been introduced. Third, while we tried our best to distinguish underlying ICAS from vessel injury after EVT, it might be difficult to distinguish them completely, which can lead to errors. Nevertheless, because these errors were applied to both groups equally, there would be no significant bias in comparing the results. Finally, in this study, the device was selected based on the practitioner’s personal preference, and there could be a bias because this was not a randomized trial. Despite these limitations, our study may be helpful in selecting devices in patients who are predicted to have ICAS-LVO [13–15].

## Conclusions

First-line SR thrombectomy showed higher immediate reperfusion and lesser vessel injury than CA for ICAS-related occlusions. However, there was no significant difference in the final reperfusion status or 3-month outcomes from additional rescue treatments. Our study may be useful for device selection in ICAS-LVO patients and warrants future large-scale prospective studies.

## Supplementary information

**Additional file 1: Supplemental Table 1.** Multivariate analysis according to iatrogenic dissection or rupture. **Supplementary figure.** (A) The first patient shows flap in the right middle cerebral artery. We detached stent retriever because of recurrent occlusion after retrieving stent retriever. (B) and (C) The second and third patients show intima flap in the left middle cerebral artery after contact aspiration and (D) The fourth patient shows intima flap in the left middle cerebral artery after retrieving stent retriever.

## Data Availability

The data of this study are available from the corresponding author upon reasonable request.

## Abbreviations

CA: Contact aspiration
EVT: Endovascular treatment
ICAS: Intracranial atherosclerotic stenosis
ICAS-LVO: Intracranial atherosclerosis-related large vessel occlusion
mRS: modified Rankin scale
mTICI: modified treatment in cerebral ischemia
NIHSS: National Institutes of Health Stroke Scale
SR: Stent retriever
