# Supplementary material for: Immediate effects of first-line thrombectomy devices for intracranial atherosclerosis-related occlusion: stent retriever versus contact aspiration
Source: BMC Neurol. 2020 Jul 18;20:283. doi: 10.1186/s12883-020-01862-6 (PMC7368707; doi:10.1186/s12883-020-01862-6)
Supplement: Supplementary file 1 — Additional file 1: Supplemental Table 1. Multivariate analysis according to iatrogenic dissection or rupture. Supplementary figure. (A) The first patient shows flap in the right middle cerebral artery. We detached stent retriever because of recurrent occlusion after retrieving stent retriever. (B) and (C) The second and third patients show intima flap in the left middle cerebral artery after contact aspiration and (D) The fourth patient shows intima flap in the left middle cerebral artery after retrieving stent retriever. [file 12883_2020_1862_MOESM1_ESM.docx]

**SUPPLEMENTARY MATERIAL**

**SUPPLEMENTARY TABLES AND FIGURES**

**Supplemental Table 1.** Multivariate analysis according to iatrogenic dissection or rupture

|  | Univariate analysis | | | Multivariate analysis | |
| --- | --- | --- | --- | --- | --- |
|  | No dissection  (N=89) | Dissection  (N=22) | P value | Odds ratio (95% CI) | P value |
| Demographics |  |  |  |  |  |
| Age, years | 64.6±13.3 | 67.7±13.8 | 0.350 | 1.027 (0.979–1.083) | 0.291 |
| Sex, men | 58 (65.2) | 12 (54.5) | 0.498 | 0.536 (0.172–1.654) | 0.275 |
| Risk factors |  |  |  |  |  |
| Hypertension | 56 (62.9) | 15 (68.2) | 0.832 |  |  |
| Diabetes mellitus | 28 (31.5) | 6 (27.3) | 0.902 |  |  |
| Dyslipidemia | 28 (31.5) | 7 (31.8) | >0.999 |  |  |
| Atrial fibrillation | 23 (25.8) | 1 (4.5) | 0.040 | 0.077 (0.004–0.494) | 0.024 |
| Smoker | 32 (36.0) | 7 (31.8) | >0.999 |  |  |
| Initial occlusion site |  |  | >0.999 |  |  |
| Internal carotid artery | 11 (12.4) | 3 (13.6) |  |  |  |
| Middle cerebral artery | 60 (67.4) | 15 (68.2) |  |  |  |
| Vertebral/basilar artery | 18 (20.2) | 4 (18.2) |  |  |  |
| Initial NIHSS score | 15 (11–20) | 17 (14–22) | 0.278 | 1.010 (0.927–1.102) | 0.820 |
| ASPECTS score* | 7 (5–9) | 8 (6–9) | 0.467 |  |  |
| Onset to puncture time, min | 325 (226–560) | 296 (242–554) | 0.552 |  |  |
| Puncture to final angiography time, min | 65 (44–98) | 82.5 (66–119) | 0.075 | 1.006 (0.994–1.017) | 0.327 |
| Laboratory findings |  |  |  |  |  |
| Hemoglobin, g/dL | 14.0±1.8 | 14.1±2.2 | 0.913 |  |  |
| White blood cells, ×10^9^/L | 10.3±4.4 | 10.9±5.7 | 0.645 |  |  |
| Platelets, ×10^9^/L | 239±67 | 242±80 | 0.893 |  |  |
| Glucose, mmol/L | 8.4±3.3 | 7.9±3.3 | 0.491 |  |  |
| IV tPA | 40 (44.9) | 10 (45.5) | >0.999 |  |  |
| First-line device |  |  | 0.012 |  |  |
| Stent retriever | 45 (50.6) | 4 (18.2) |  | Ref |  |
| Contact aspiration | 44 (49.4) | 18 (81.8) |  | 4.488 (1.394–17.676) | 0.018 |
| Tirofiban infusion | 38 (42.7) | 14 (63.6) | 0.128 |  |  |
| Successful reperfusion after the 1^st^ attempt | 55 (61.8) | 10 (45.5) | 0.249 |  |  |
| Final successful reperfusion | 75 (84.3) | 16 (72.7) | 0.223 |  |  |
| Number of techniques | 2 (1–3) | 2 (2–3) | 0.045 | 1.094 (0.519–2.326) | 0.812 |

* Baseline ASPECTS on quality imaging was evaluated in 81 patients (91.0% patients with anterior circulation infarction).

NIHSS: National Institutes of Health Stroke Scale; IV tPA: intravenous tissue plasminogen activator

**Supplementary figure 1 and its legend**


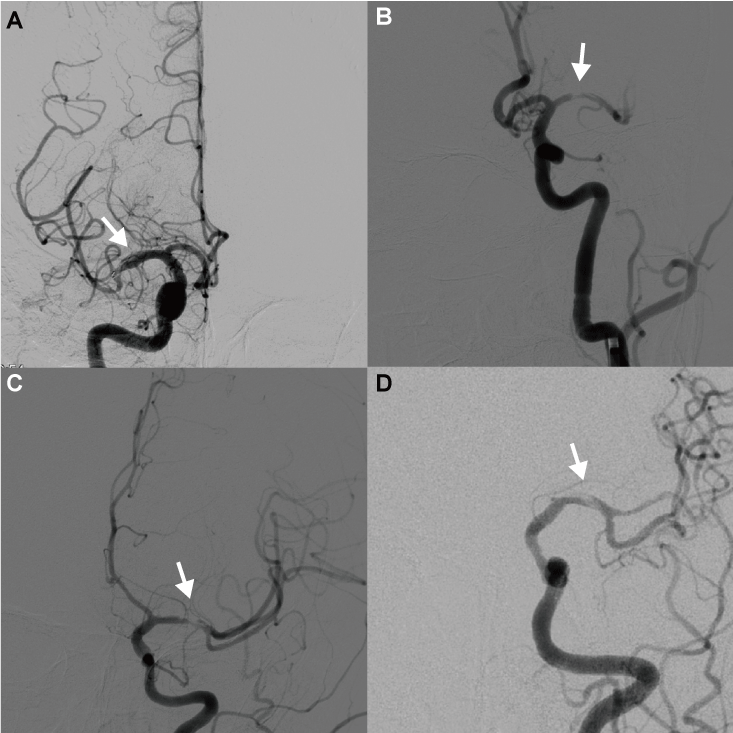


**Supplementary figure.** (A) The first patient shows flap in the right middle cerebral artery. We detached stent retriever because of recurrent occlusion after retrieving stent retriever. (B) and (C) The second and third patients show intima flap in the left middle cerebral artery after contact aspiration and (D) The fourth patient shows intima flap in the left middle cerebral artery after retrieving stent retriever.
